# Babesiosis in Immunocompetent Patients, Europe

**DOI:** 10.3201/eid1701.100737

**Published:** 2011-01

**Authors:** Martin Martinot, Mahsa Mohseni Zadeh, Yves Hansmann, Isabelle Grawey, Daniel Christmann, Sarah Aguillon, Maggy Jouglin, Alain Chauvin, Dominique De Briel

**Affiliations:** Author affiliations: Hôpitaux Civils De Colmar, Colmar, France (M. Martinot, M.M. Zadeh, I. Grawey, S. Aguillon, D. De Briel);; Hôpitaux Universitaires de Strasbourg, Strasbourg, France (Y. Hansmann, D. Christmann);; École Nationale Vétérinaire Agroalimentaire et de l’Alimentation Nantes-Atlantique, Nantes, France (M. Jouglin, A. Chauvin);; Institut National de la Recherche Agronomique, Nantes (M. Jouglin, A. Chauvin)

**Keywords:** Babesia, Anaplasma, babesiosis, tick-borne infection, parasite, zoonoses, immunocompetent, Europe, dispatch

## Abstract

We report 2 cases of babesiosis in immunocompetent patients in France. A severe influenza-like disease developed in both patients 2 weeks after they had been bitten by ticks. Diagnosis was obtained from blood smears, and *Babesia divergens* was identified by PCR in 1 case. Babesiosis in Europe occurs in healthy patients, not only in splenectomized patients.

Babesiosis, a tick-borne infectious disease that occurs worldwide, is caused by species of *Babesia*, an intraerythrocytic parasite (*1*). *Babesia* spp. parasites infect wild and domesticated animals and may cause a malaria-like syndrome. The first human case was described in 1957 in a splenectomized Yugoslavian farmer who died (*2*). More than 100 *Babesia* species infect animals, but human infection has been associated with only a few species, mainly *B. microti* and *B. divergens* (*1*–*3*). *B. microti* parasites are transmitted by *Ixodes scapularis* ticks and infect rodents. Since 1957, these parasites have caused hundreds of human babesiosis cases in the United States, the most affected country. Infections are found mainly in healthy persons and manifest as asymptomatic or mild to moderate illness; severe disease, even in immunocompromised or elderly patients, is seldom reported (*2*,*3*). *B. divergens* parasites are endemic to Europe; they are transmitted by *I. ricinus* ticks and infect bovines (*4*). In Europe, the disease is rare in humans; ≈40 cases have been reported (*2*,*3*,*5*–*7*). These cases are almost exclusively severe in immunocompromised patients, especially those whose spleens have been removed (*2*,*3*,*8*). *B. divergens* parasites are responsible for >70% of these cases (*2*,*8*), although the disease is not always confirmed by molecular-based methods.

We report 2 cases of human babesiosis in Colmar, Alsace, a northeastern region of France in which Lyme disease is endemic. The disease was diagnosed in spring 2009 in healthy young persons without history of travel abroad who experienced a marked influenza-like syndrome and recovered. These cases should change the classic description of babesiosis in Europe, in which the disease was thought to affect immunocompromised patients exclusively. Our study indicates that this disease also occurs in Europe among immunocompetent patients.

## Case Reports

Patient 1, a 37-year-old woman without known medical history, sought treatment on April 29, two weeks after a tick bite. She had a 38.5°C fever with chills, headaches, and arthromyalgia. Results of a physical examination were normal. Laboratory findings included leukopenia (3,300 leukocytes/µL, 45% polymorphonuclear cells, 37% lymphocytes), aspartate aminotransferase and alanine aminotransferase levels of 136 IU/L and 160 IU/L, respectively; γ-glutamyl transpeptidase 135 IU/L; alkaline phosphatase 131 IU/L; and elevated C-reactive protein level (48 mg/L). Serologic results for Lyme disease, tick-borne encephalitis virus, tularemia, *Anaplasma* spp*.*, *Coxiella burnetii*, and *Rickettsia* spp*.*, as well as blood cultures were negative.

**Figure 1:**
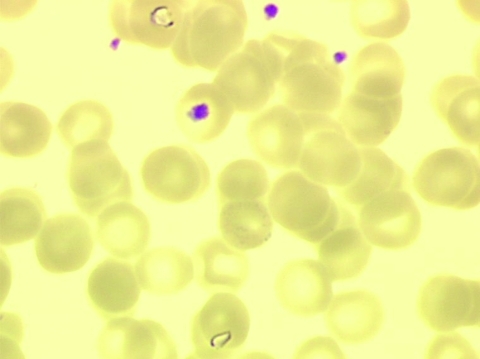
Two trophozoites, pear-shaped, of *Babesia divergens* in erythrocytes from case-patient 1 (original magnification ×1,000, May-Grünwald-Giemsa stain).

A thin peripheral blood smear stained with May-Grünwald-Giemsa did not show any ehrlichial morulae in granulocytes, as suspected, but a retrospective examination of stored slides on May 22 (the same day that case 2 was characterized) showed pear forms and trophozoites of intraerythrocytic parasites (parasitemia level 0.29%), leading to the diagnosis of babesiosis (Figure 1). The patient had initially received doxycycline (200 mg/d) for a suspected bacterial tick-borne infection, and her symptoms rapidly resolved. The first blood sample was discarded, but on June 16 and June 24, additional serum and whole blood samples were collected in sodium citrate vacutainer tubes (Becton-Dickinson, Franklin Lakes, NJ, USA). The blood smears remained positive until July but were negative in August.

Patient 2, a 35-year-old man with an uneventful medical history, was hospitalized on May 21, two weeks after receiving 3 tick bites. He had a 39°C fever, severe headaches, and arthromyalgia. Results of a physical examination were normal. Laboratory findings showed leukopenia (1,860 leukocytes/µL, with 35% polymorphonuclear leukocytes, 49% lymphocytes), marked thrombopenia with 36,000 platelets/mm^3^, elevated liver enzyme levels (aspartate aminotransferase 70 IU/L, alanine aminotransferase 77 IU/L, γ-glutamyl transferase 161 IU/L, and alkaline phosphatase 86 IU/L), and elevated C-reactive protein (124 mg/L). Tick-transmitted disease serologic results and blood cultures were negative.

**Figure 2:**
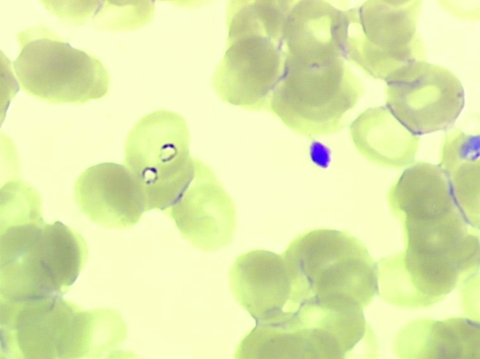
Two trophozoites of *Babesia* spp. in 1 erythrocyte from case-patient 2 (original magnification ×1,000, May-Grünwald-Giemsa stain).

A thin peripheral blood smear stained with May-Grünwald-Giemsa showed intraerythrocytic *Babesia* spp. (parasitemia level 0.23%) (Figure 2). The patient received azithromycin 500 mg on day 1 then 250 mg/day plus atovaquone, and his illness rapidly resolved. Two samples of serum and whole blood were collected in sodium citrate vacutainer tubes on June 16 and July 21 and sent to the veterinary laboratory of Nantes for *B. divergens* serologic analysis (indirect immunofluorescent assay by using the gerbil-derived strain *B. divergens* Rouen F5 antigen), erythrocyte cultures, and DNA extraction (Wizard genomic DNA Purification kit; Promega, Madison, WI, USA) for PCR *Babesia* spp (*9*). .

Serologic results and cultures remained negative for both patients. However, serologic analysis is neither sensitive nor specific (*7*,*10*), and cultures probably were inhibited because blood samples were collected after doxycycline or azithromycin proguanil treatments. The PCR for *Babesia* spp. is specific for an 18S rDNA 540-base long region of a variable part of the gene with Bab primers GF2 and GR2, was performed (*9*,*11*). Results were positive for patient 1. The sequencing of PCR products showed 100% homology with *B. divergens* human strains GenBank accession nos. FJ944822 and FJ944823 (*9*). PCR results were negative for patient 2. Samples from patient 2 were collected 1 month after treatment with atovaquone-proguanil, and the blood smear was negative. However, the clinical and biological data and the observation of trophozoites (especially 2 trophozoites in 1 erythrocyte) in the blood smear from patient 2 confirmed by a reference laboratory led us to strongly suspect babesiosis (Figure 2). In this case, the *Babesia* species remains unknown, and a non–*B. divergens* species cannot be ruled out, although it is rarer.

## Conclusions

Our cases highlight that, in Europe, babesiosis can occur in healthy persons and manifest as moderate illness. The rarity of other reported cases in nonimmunocompromised patients in Europe may be related to the difficulty of diagnosing babesiosis. A stained thin blood smear is rarely performed in Europe after tick bite in healthy patients. The difficulty of detecting intra-erythrocytic forms of babesia coupled with frequent low levels of parasitemia, may result in accurate diagnoses, although acridine orange and fluorescent microscopy may assist in the detection of parasites (*1*). Other diagnostic tests, such as PCR and serologic analysis, are not routinely performed in France and require a reference laboratory (*8*).

Babesiosis, although difficult to diagnose, needs to be diagnosed for various reasons: 1) without treatment, babesiosis can lead to severe illness; 2) the disease can persist for a long period without symptoms, which could lead to posttransfusion cases (*12*); and 3) effective specific treatments are available (atovaquone plus azithromycin, or for severe cases, clindamycin and quinine) (*2*). These drugs are not usually prescribed in febrile tick-bite cases; doxycycline is the usual drug used to treat tick-borne bacterial diseases. Moreover, patients with moderate infection could benefit from an atovaquone plus azithromycin regimen, which is better tolerated (*13*).

Previous serosurveys from tick-exposed patients or healthy blood donors in France (*7*), Germany (*14*), and Switzerland (*15*) have demonstrated antibodies against *Babesia* spp. antigens ranging from 1.0% to 11.5%. These data suggest that *Babesia* spp. infections probably occur more frequently in Europe than previously believed and may affect healthy patients. Although most patients may be asymptomatic, our 2 cases demonstrate that babesiosis can result in a serious influenza-like syndrome in previously healthy patients. In Europe, babesiosis is probably underdiagnosed; thus, we suggest that when patients have influenza-like or malaria-like syndromes after confirmed or suspected tick bites, a blood smear be performed regardless of whether the patient is immunocompromised. Blood smear can identify not only *Babesia* spp. infection but also *Anaplasma* spp. infection*,* another emerging and underdiagnosed tick-borne illness. In cases of new European *Babesia* spp. infections, a deeper characterization of the strains by erythrocytes cultures and standardized PCR, as well as a systematic study of the patients’ immune systems, should be undertaken to enable a better understanding of this disease.
